# Male Circumcision and Sexual Risk Behaviors May Contribute to Considerable Ethnic Disparities in HIV Prevalence in Kenya: An Ecological Analysis

**DOI:** 10.1371/journal.pone.0106230

**Published:** 2014-08-29

**Authors:** Chris Richard Kenyon, Lung Vu, Joris Menten, Brendan Maughan-Brown

**Affiliations:** 1 HIV/STI Unit, Institute of Tropical Medicine, Antwerp, Belgium; 2 Division of Infectious Diseases and HIV Medicine, University of Cape Town, Cape Town, South Africa; 3 Population Council, Washington, D.C., United States of America; 4 Southern Africa Labour and Development Research Unit, University of Cape Town, Cape Town, South Africa; University of New South Wales, Australia

## Abstract

**Background:**

HIV prevalence varies between 0.8 and 20.2% in Kenya’s various ethnic groups. The reasons underlying these variations have not been evaluated before.

**Methods:**

We used data from seven national surveys spanning the period 1989 to 2008 to compare the prevalence of a range of risk factors in Kenya’s ethnic groups. Spearman’s and linear regression were used to assess the relationship between HIV prevalence and each variable by ethnic group.

**Results:**

The ethnic groups exhibited significant differences in a number of HIV related risk factors. Although the highest HIV prevalence group (the Luo) had the highest rates of HIV testing (Men 2008 survey: 56.8%, 95% CI 51.0–62.5%) and condom usage at last sex (Men 2008∶28.6%, 95% CI 19.6–37.6%), they had the lowest prevalence of circumcision (20.9%, 95% CI 15.9–26.0) the highest prevalence of sex with a non-married, non-cohabiting partner (Men: 40.2%, 95% CI 33.2–47.1%) and pre-marital sex (Men 2008∶73.9%, 95% CI 67.5–80.3%) and the youngest mean age of debut for women (1989 Survey: 15.7 years old, 95% CI 15.2–16.2). At a provincial level there was an association between the prevalence of HIV and male concurrency (Spearman’s rho = 0.79, P = 0.04). Ethnic groups with higher HIV prevalence were more likely to report condom use (Men 2008 survey: R2 = 0.62, P = 0.01) and having been for HIV testing (Men 2008 survey: R2 = 0.47, P = 0.04).

**Conclusion:**

In addition to differences in male circumcision prevalence, variation in sexual behavior may contribute to the large variations in HIV prevalence in Kenya’s ethnic groups. To complement the prevention benefits of the medical male circumcision roll-out in several parts of Kenya, interventions to reduce risky sexual behavior should continue to be promoted.

## Introduction

The average HIV prevalence in Kenya in 2008 was 7% and varied between 0.8 and 20.2% in Kenya’s various ethnic groups (aged 15–49 years) [1] and were similar to those in 2003. There is little published that attempts to investigate what factors are responsible for these differences [2]–[5]. This is an important question to answer since elucidating the contributing factors may assist with efforts to further decrease HIV incidence.

In this study we use a number of nationally representative data sources from early to late in the HIV epidemic (1989 to 2011) to compare the prevalence of various HIV risk factors between different Kenyan ethnic groups. The risk factors investigated include measures of sexual network connectivity. Since HIV is transmitted via networks of sexual partnerships, the differences in the structure of these networks may be important determinants of the prevalence of HIV in a particular population. Differences in sexual network connectivity have been shown to play an important role in explaining the racial/ethnic differences in STI prevalence elsewhere [6]–[10]. The extent to which this is true in Kenya is investigated.

An important preliminary question is whether sexual relationships in Kenya exhibit ethnic homophilous partnering. Ethnic homophily in choosing sex partners is necessary for investigations of sexual networks and risk factors to be meaningful [11]. Data from Kisumu suggest that ethnic homophily is important in partner choice [11]. In this study we therefore first assess whether this is also the case in the rest of Kenya and then, given that we establish it is, we examine HIV risk factors by ethnic group.

**Figure 1:**
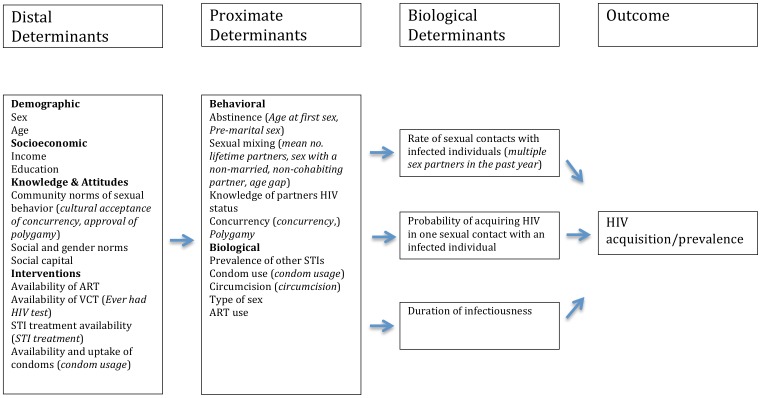
Conceptual framework for understanding the determinants of HIV acquisition/prevalence (based on the work of Boerma et al., [12] and Barnighausen et al [13]. (*The names of the variables used in the current study are indicated inside parentheses*).

An appropriate theoretical framework is crucial to systematically examine how different HIV risk factors are connected via specific causal pathways that determine HIV infection. The conceptual framework we use to interpret the relationship between the various putative determinants of HIV acquisition and prevalence is the proximate-determinants approach as developed by Boerma et al [12] and Barnighausen et al [13] (see Figure 1).

## Methodology

We used seven nationally representative data sets for this analysis. The research involved secondary data analysis of these surveys that had each received ethical committee clearance for data analyses such as the one performed here. All data is aggregated to the level of communities and thus anonymity is preserved. No specific ethics committee approval was necessary for this study. The detailed methodologies of these surveys have been described elsewhere but are summarized below [1], [14]–[18].

The WHO/Global Programme on AIDS (GPA) conducted a sexual behavioral survey in Kenya in July 1989 to February 1990 [14]. A two-stage cluster sampling was used with enumeration areas as the first stage and households as the second. This yielded a nationally representative sample of 2691 Kenyan 15–59 years old - excluding the North Eastern province that was not included in the survey. We limited the analysis to those between 15 and 49 years age –1168 men and 1523 women.

Five Kenya Demographic and Health Surveys (KDHS) conducted in 1989, 1993, 1998, 2003 and 2008 were utilized. The 2007 and 2012 Kenya AIDS Indicator Surveys were not used as they were not publically available and they did not collect information on the respondent’s ethnicity. Each KDHS utilized a household-based, two-stage stratified sampling approach which once weighted provides prevalence estimates that are representative for the provinces sampled. The first stage selected sample points from a national master sample maintained by Kenya’s Central Bureau of Statistics. The second stage involved the systematic sampling of a household list. In each selected house all women aged 15–49 who slept in that house the previous night were selected. In addition, in every second house all men aged 15–54 were selected to complete the survey. In the 2003 and 2008 survey all eight provinces were sampled, but in the first three surveys all three districts in the Northeastern Province and four other northern districts (Isiolo and Marsabit from Eastern Province and Samburu and Turkana from the Rift Valley Province) were excluded. Together these excluded districts account for less than four percent of Kenya's population [15]. In the 2003 and 2008 surveys, in all households selected for the Men’s Questionnaire, a finger prick blood specimen was collected from all consenting, eligible male and female participants.

The Population Services International (PSI) Survey/Kenya 6th HIV Survey conducted in 2011 used a two stage cluster sampling to obtain a provincially representative sample of households from seven of Kenya’s eight provinces (the North East was excluded). In each selected household all 15–49 year old men and women were eligible for participation. The ethnicity of the respondents was not reported. Ethnic group membership in Kenya is highly correlated with region of residence [1]. For example, 75.1% of the Luo and 76.3% of the Kisii live in Nyanza Province where they make up 60.1 and 32.1% of the population, respectively (authors calculations of KDHS 2008 data). Further details of the surveys are provided in Table 1.

**Table 1:** Characteristics of the seven surveys used in the study.

	1989 GPA	1989 DHS	1993 DHS	1998 DHS	2003 DHS	2008 DHS	2011 PSI
Sample size men^a^	1168	1129	2762	3845	4183	3910	1805
Sample size women^a^	1523	7150	7952	8233	8717	8767	1246
Household response rate	NS	98.0	97.1	96.8	96.3	97.7	NS
Overall women’s response rate^b^	NS	96.3	92.1	92.6	90.1	94.1	NS
Overall men’s response rate^b^	NS	80.8	82.1	85.6	82.4	86.6	NS
HIV testing	No	No	No	No	Yes	Yes	No

Abbreviation: NS - Not Specified.

a Unweighted.

b The overall women’s/men’s response rate is defined as the eligible women’s/men’s response rate x the household response rate (see report for details).

### HIV

Dried blood samples were collected and subsequently tested for HIV using the Vironostika Anti-HIV-1/2 Plus enzyme-linked immunosorbent assay (ELISA) test kit. All positive samples and 5 percent of negative samples were retested with a Murex HIV-1/2 MicroELISA System. HIV tests were conducted for 83 percent of eligible respondents, including 86 percent of the 4,418 eligible women and 79 percent of the 3,910 eligible men. Response rates did differ somewhat by region (for details see [2]). In this paper we use HIV prevalence by ethnic group in the 2008 KDHS as the outcome variable. HIV prevalence by ethnic group is defined as the percent of all persons 15–49 year old who test HIV positive out of all those tested for HIV.

### Ethnicity

Ethnicity was measured by self-reporting. Respondents in the KDHS, for example, were asked, “What is your ethnic group/tribe?”.

### Homophily

The available datasets only collected information about the ethnicity of live–in-husbands or partners. As a result, the degree of ethnic-homophily per ethnic group was defined as the percentage of married women whose husbands or live in partners were from the same ethnic group. These figures were calculated from the 1989 and 2008 Demographic and Health Surveys (DHS).

Each of following predictor variables were calculated separately for men and women and were limited to those between the ages of 15 and 49 years. The denominator used for all prevalence figures excluding HIV prevalence, premarital sex, circumcision and cultural acceptance of concurrency was limited to those individuals who reported ever having had sex.

#### Sex with a non-married, non-cohabiting partner

The percentage who had sex with a non-marital, non-cohabiting partner in the past 12 months, among those who have had sex in the past 12 months.

#### Multiple sex partners in past year

The percentage who had two or more sexual partners in the past 12 months, among those who have had sex in the past 12 months. In the 1993 survey this could only be assessed over the prior 6 months.

#### Age at first sex

The mean age of reported first sexual intercourse. This is calculated for those aged 15–24 and 15–49 years.

#### Pre-marital sex

The percent of never-married 15–24 year olds respondents who reported having had sex.

#### Condom usage

This was assessed in various ways in the different surveys.

#### Ever used a condom

The percentage who reported ever having used a condom before. This variable is taken from the GPA survey and is used to assess condom usage early on in the epidemic.

#### Condom use at last sex

The percentage who reported using a condom at last sex.

#### Male circumcision

The percent of men who reported being circumcised.

#### STI treatment

The percent who obtained STI treatment at a medical centre among those who reported having STI symptoms in the past 12 months. Due to the low numbers who reported STIs this variable is reported for men and women combined.

#### Polygamy

The percentage of married women who report that their husband has more than one wife or partner at the time of the survey. This could not be defined from the men’s questionnaires as the men were asked ‘how many wives *or partners* do you currently have?’.

#### Cumulative prevalence of concurrency

The percentage of respondents who had two or more sex partners at the same time at any point in the past 12 months. This could only be calculated from the PSI survey, which asked two partner-specific questions for the last five sexual partners in the prior 12 months: “When did you last have sex with this partner?” and “When did you first have sex with this partner?”.

#### Cultural acceptance of male concurrency

This was calculated from the question in the PSI survey: “Is it culturally acceptable for men to have more than one partner?” We dichotomized the answers into those who agreed or strongly agreed and those who disagreed or strongly disagreed.

#### Approval of polygamy

The percentage of sexually active men who reported that they generally approve of polygamy. This was based on a question in the 1989 KDHS that was only directed to men: “Generally, do you approve or disapprove of polygamy?” Responses were coded dichotomously.

### Statistical analysis

All analyses are ecological in nature and conducted with HIV prevalence by ethnic group in KDHS 2008 as the dependent variable. The analyses were conducted using STATA 12.0 (College Station, TX). All standard errors have been adjusted to account for the complex sampling strategies of the six surveys using the survey (SVY) command. Only ethnic groups that constituted more than 2% of the population survey were included in the analyses. The analyses were stratified by gender. The strength and direction of the relationship between HIV prevalence and the majority of the HIV risk factors from different surveys were assessed using Pearson product-moment correlation. The relationship between concurrency and HIV prevalence in the PSI survey was non-linear and thus Spearman’s correlation was used to assess the relationship between HIV prevalence and the variables from this study. The statistical significance of the relationships were assessed using bi-variate regression analysis. In sensitivity analyses we repeated our regression analyses with (1) the HIV prevalence rates by ethnic group from the 2003 KDHS as the dependent variable; and (2) the average of the 2003 and 2008 KDHS’s HIV prevalence rates by ethnic group as the dependent variable.

## Results

An overview of the number and mean age of men and women by ethnic group participating in each of the surveys is provided in Table 2. The average age of the samples in all studies ranged from 27.1 to 30.2. The HIV prevalence by ethnic group in 2008 varied between 0.8% (95% CI, 0.3–1.8%) in the Somali and 20.3% in the Luos (95% CI, 16.4–26.2%; see Table 3). With the exception of the Taita/Taveta the HIV prevalence estimates produced by the two surveys were very similar. This variation may be due to the fact that the Taita/Taveta had the smallest HIV testing sample size of all the surveyed ethnic groups (72 individuals in 2008). The overall HIV testing response rates were 73% in the 2003 KDHS and 83% in the 2008 DHS (See Table 3). Of those who were not tested in 2008, 11% refused HIV testing, 4% were not at home for the testing and in 2% there were problems with the obtaining consent or blood specimens. Response rates varied by ethnic group ranging from 74.2% in the Somali to 95.5% in the Kisii in 2008.

**Table 2:** Sample sizes, mean ages and distribution of the wealth and educational attainment of the ethnic groups in the surveys.

	Somali	Kalenjin	Taita/Taveta	Mijikenda/Swahili	Kamba	Kikuyu	Kisii	Meru	Luhya	Luo
**GPA**										
Mean age (95% CI)^a^		30.2 (29.0–31.3)			28.3 (27.2–29.5)	29.9 (29.1–30.6)	28.0 (26.3–29.7)		29.5 (28.4–30.5)	28.7 (27.7–29.7)
Women n^a^		137			167	367	68		168	177
Men n^a^		111			88	301	45		139	144
**1989**										
Mean age (95% CI)		29.0 (28.2–29.8)		29.9 (28.1–31.7)	28.2 (27.5–28.9)	28.5 (27.6–29.4)	28.0 (27.3–28.7)	29.4 (28.4–30.4)	28.1 (27.5–28.7)	27.9 (27.2–28.6)
Women n		617		469	686	1735	449	380	1247	1151
**1993**										
Mean age (95% CI)		29.3 (28.4–30.2)	29.2 (28.0–30.4)	28.6 (27.8–29.3)	29.5 (28.9–30.2)	29.1 (28.6–29.5)	28.4 (27.5–29.2)	30.0 (29.2–30.8)		28.6 (28.1–29.1)
Women n		1096	276	527	761	1506	549	438		933
Men n		379	65	164	221	452	159	176		261
**1998**										
Mean age (95% CI)		28.5 (27.7–29.2)	28.8 (27.9–29.8)	28.4 (27.9–28.9)	28.2 (27.6–28.7)	28.8 (28.3–29.2)	27.1 (26.6–27.6)	28.6 (27.8–29.5)	28.9 (28.4–29.4)	28.3 (27.6–29.0)
Women n		1316	291	633	855	1255	645	503	1117	959
Men n		549	135	234	385	522	255	253	518	404
**2008**										
Mean age (95% CI)	28.2 (27.5–28.9)	28.4 (27.5–29.3)		28.0 (27.2–28.9)	28.9 (28.3–29.6)	30.2 (29.5–30.9)	28.5 (27.8–29.2)	30.2 (29.5–30.9)	28.5 (27.9–29.0)	27.4 (26.7–28.0)
Women n	679	750		717	666	1504	447	367	1266	1113
Men n	223	316		257	286	584	188	172	566	486
**Wealth Quintiles** b										
Poorest	56.6	31.3	0.2	31.3	16.4	1.7	11.0	5.8	11.7	13.4
Poorer	4.9	26.3	9.2	14.1	17.4	5.7	26.3	15.1	24.6	19.4
Middle	5.8	18.8	15.9	8.1	21.9	17.2	21.5	33.0	19.5	16.9
Richer	6.5	16.7	26.7	19.7	22.7	27.9	18.2	30.0	16.5	20.7
Richest	26.1	6.9	48.1	26.9	21.6	47.5	23.1	16.2	27.8	29.5
**Education** c										
No education	64.9	7.8	7.0	25.0	2.6	1.2	1.3	2.9	3.0	1.6
Primary	20.9	60.6	41.2	54.6	62.4	49.0	43.9	63.8	61.4	63.6
Secondary	10.8	25.3	33.5	17.3	26.9	38.5	46.7	26.6	28.2	26.7
Post Secondary	3.4	6.3	18.3	3.2	8.1	11.3	8.1	6.8	7.3	8.1

Only the ethnic groups which were used in the analysis are shown. All ethnic groups whose sample size constitutes less than 2% of the total sample size were dropped.

a Sample sizes are unweighted but the mean age figures are weighted results.

b Wealth quintiles are derived from an asset index in the 2008 KDHS (see reference for details [1]).

c Highest education level attained: Primary and Secondary refer to any primary or secondary schooling attained respectively. The data is from the 2008 KDHS.

**Table 3:** HIV prevalence and HIV testing response rates in 15–49 year olds by ethnic group in 2003 and 2008 Kenyan Demographic and Health Surveys.

	2003 Survey			2008 Survey		
	HIV Prevalence-% (95% CI)	n	Response Rate^a^	HIV Prevalence- % (95% CI)	n	Response Rate^a^
**Somali**	1.3 (0.0–2.6)	177	72.5	0.8 (0.3–1.8)	171	74.2
**Kalenjin**	3.4 (1.0–5.7)	712	89.4	1.8 (0.3–3.1)	929	91.4
**Taita/Taveta**	9.7 (1.8–17.6)	71	88.1	3.3 (0.2–6.4)	72	93.8
**Mijikenda/Swahili**	3.5 (1.7–5.2)	254	81.2	3.3 (1.2–5.4)	316	92.9
**Kamba**	4.9 (2.8–8.0)	1363	79.1	4.0 (2.3–5.6)	1246	88.4
**Kikuyu**	5.4 (3.7–6.1)	726	73.5	4.6 (2.7–6.4)	750	89.8
**Kisii**	4 (1.6–6.5)	334	94.1	4.6 (2.4–6.8)	471	95.5
**Meru**	3.7 (1.4–6.0)	337	77.7	5.4 (0.9–10.0)	339	94.6
**Luhya**	6.6 (4.2–9.0)	919	87.9	7.0 (4.5–9.6)	1096	91.3
**Luo**	21.8 (17.7–25.9)	702	88.1	20.3 (16.4–24.2)	868	92.5

a The HIV testing response rate is defined as the percentage of eligible persons 15–49 years old who participated in HIV testing. Non-responders included those who refused testing, were absent at the time of the survey or there were technical difficulties with blood taking.

### Homophiliy

Ethnic-homophily rates for married couples were high in all ethnic groups. In 1989 all groups had homophily rates above 89% and the median homophily rate was 95.4% (see Table 4). Repeating this analysis stratified by province made little difference. Even in Nairobi, homophily rates remained high –88 to 100% in all ethnic groups represented by more than four individuals (results not shown). In the 2008 survey, the homophily rates varied between 62.5 in the Taita (the numerically smallest group) and 97.1% in the Somali. The median homophily rate was 87% (results not shown).

**Table 4:** Homophily by ethnicity.

	Husband’s ethnicity										
Wife’s ethnicity	Kalenjin	Kamba	Kikuyu	Kisii	Luhya	Luo	Meru	Mijikenda/Swahili	Somali	Other	Total
**Kalenjin**	119 (98.4)	0	0	0	1(0.8)	0	0	0	0	1 (0.8)	121
**Kamba**	1 (0.8)	116 (98.3)	1 (0.8)	0	0	0	0	0	0	0	118
**Kikuyu**	2 (0.8)	2 (0.8)	249 (95.4)	0	3 (1.2)	1 (0.4)	1 (0.4)	1 (0.4)	0	2 (0.8)	261
**Kisii**	0	0	2 (3.4)	56 (94.9)	0	0	0	0	0	1 (1.7)	59
**Luhya**	3 (1.8)	1 (0.6)	0	0	151 (89.4)	9 (5.3)	0	0	0	5 (3.0)	169
**Luo**	0	1 (0.6)	0	1 (0.6)	5 (2.8)	169 (94.9)	0	0	0	2 (1.1)	178
**Meru**	0	1 (1.2)	2 (2.4)	0	0	0	79 (96.3)	0	0	0	82
**Mijikenda/Swahili**	0	0	0	0	0	0	0	65 (92.9)	0	5 (7.1)	70
**Somali**	0	0	0	0	0	0	0	1 (33.3)	2 (66.7)	0	3
**Other**	1 (1.5)	0	3 (4.5)	0	6 (9.0)	0	0	4 (6.0)	0	53 (79.1)	67
**Total**	126	121	257	57	166	179	80	71	2	69	1128

The self-defined ethnicity of married husbands and wives in the 1989 Kenyan Demographic and Health Survey (Row percentages).

### Potential HIV risk factors

**Figure 2:**
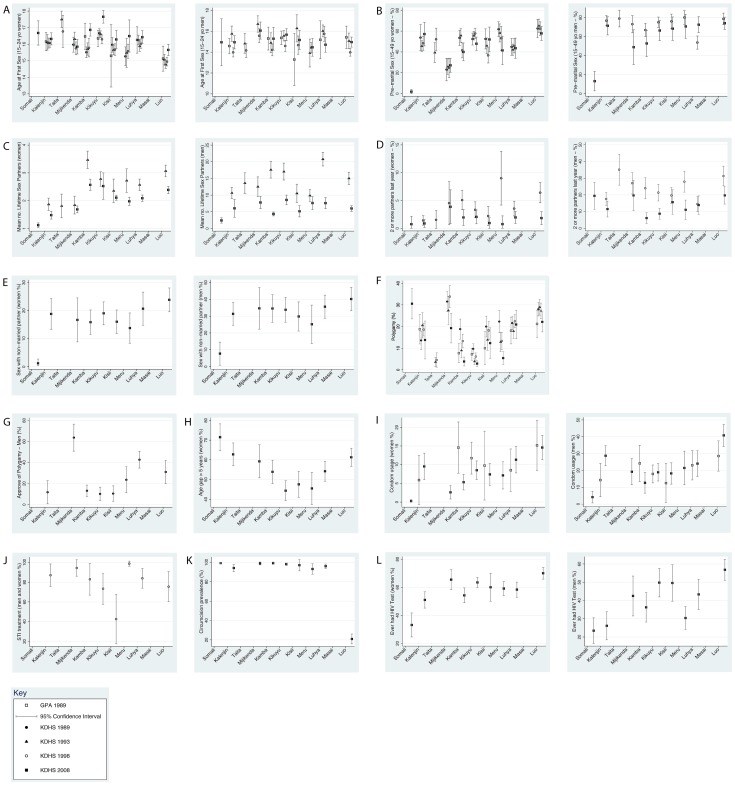
Prevalence of sexual behaviors, condom use and circumcision by ethnic group and sex in six Kenyan surveys of 15–49 year olds. Age of first sex (A), percent reporting pre-marital sex (B), mean number of lifetime sexual partners (C), percent reporting two or more sex partners in past 12 months (D), percent reporting sex with a non-married, non-cohabiting partner in prior 12 months (E), polygamy, (percent of married women who report that their husband has other wives) (F), percent of men who approve of polygamy (G), age gap – the percentage of women with a partner more than five years older than them (H), condom usage - percentage who reported ever having used a condom before (for GPA survey), and the percentage who reported using a condom at last sex (for KDHS 2008 survey - I), STI treatment - the percent who obtained STI treatment at a medical centre among those who reported having STI symptoms in the past 12 months (J), the percent of men who reported being circumcised (K), percent ever tested for HIV (L). Point estimates with 95% Confidence Intervals. KDHS (Kenya Demographic and Health Survey), GPA (Global Programme on AIDS).

**Figure 3:**
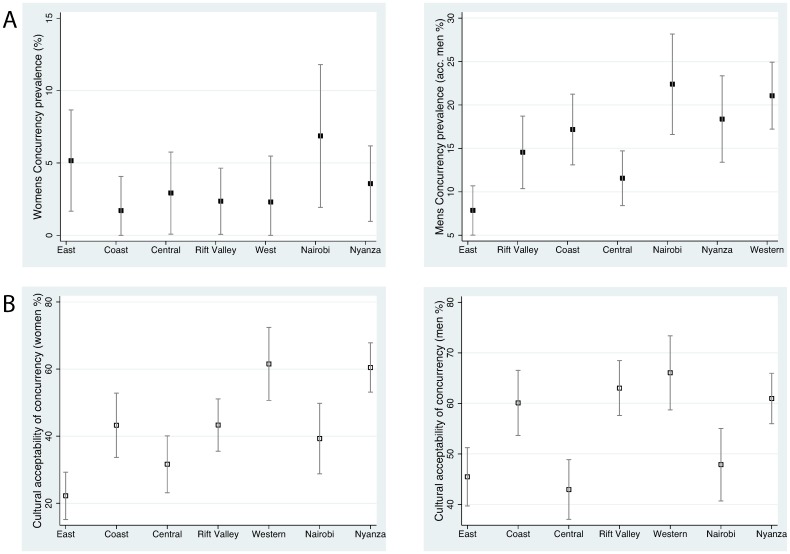
Prevalence of concurrency and acceptability of male concurrency by province. The percent who had two or more sex partners at the same time at any point in the past 12 months (A) and the percent reporting it is culturally acceptable for men to have more than one partner (B). Population Services International (PSI) Survey.

Selected descriptive statistics of the prevalence of HIV risk factors by ethnic group are presented here. Figures 2 and 3 show the prevalence of each of the potential HIV risk factors across all surveys by ethnic group and province, respectively. All results reported from the correlation analyses are summarized in Table 5.

**Table 5:** The association between HIV prevalence in 2008 and various risk factors by ethnic group in six surveys.

	Men			Women		
	n	r	P-value	n	r	P-value
**Circumcision (2008)**	9	−0.95	0.0001			
**Age at first sex (15**–**24 years old)**						
GPA	6	0.35	0.49	6	−0.68	0.14
1989				8	−0.59	0.12
1993	9	−0.17	0.68	9	−0.68	0.04
1998	9	−0.19	0.62	9	−0.73	0.02
2008	9	−0.20	0.60	9	−0.49	0.18
**Pre marital sex (15–24 years old)**						
1989				8	0.64	0.09
1993				9	0.61	0.08
1998	9	0.21	0.58	9	0.61	0.08
2008	9	0.47	0.19	9	0.59	0.09
**Proportion with 2 or more sex partners in last year**						
1998		0.28	0.58	9	0.45	0.19
2008		0.31	0.41	9	0.11	0.78
**Mean no. of lifetime sex partners**						
1993	9	0.21	0.58	9	0.48	0.18
2008	9	0.15	0.69	9	0.49	0.18
**Sex with non-married, non-cohabiting partner (2008)**	9	0.54	0.12	9	0.60	0.09
**Polygamy**						
GPA				6	0.57	0.24
1989				8	0.45	0.26
1993				9	0.52	0.15
1998				9	0.39	0.29
2008				9	0.17	0.66
**Cumulative concurrency**						
PSI^b^	7	0.78 c	0.04	7	0.25 c	0.59
**Think concurrency is culturally acceptable (PSI 2011)** b		0.33	0.46		0.69	0.08
**Approval of polygamy (1989)**	8	0.15	0.71			
**Age gap>5years (2008)**					0.00	0.99
**Condom usage**						
GPA	6	0.74	0.09		0.60	0.21
2008	9	0.78	0.012		0.75	0.02
**Those with STI went for treatment (1998)** a	9	0.12	0.77	9	0.12	0.77
**Ever tested for HIV (2008)**	9	0.68	0.04	9	0.61	0.07

a Due to the low numbers who reported having had STI symptoms in the past 12 months the analysis was only performed for men and women combined.

b As detailed in the methods section, the associations between HIV and concurrency are by Province of residence and not by ethnic group in the PSI survey.

c Spearman’s Rho correlation was used for these comparisons.

#### Male Circumcision

The Luo had a markedly lower reported circumcision prevalence (20.2%, 95% Confidence Interval: 15.9–26.0) than the other groups. Circumcision prevalence was very high in all other groups, with a range from 93.2% among the Meru to 99.3% among the Somali. Circumcision prevalence and HIV prevalence were strongly correlated (r = −0.95, P = 0.0001). This relationship was driven by the Luo who had a high HIV but a low circumcision prevalence. There was no significant relationship between circumcision and HIV prevalence when the regression was repeated without the Luo (r = −0.31, P = 0.45).

#### Age at first sex

There was little heterogeneity in the reported mean age at first sex between different ethnic groups or across time in the 15–49 year old men with the noted exception of a late age of debut amongst the Somali’s (2008 survey: 22.1, 95% CI 21.0–23.0). The group with the second latest age at first sex were the Mijikenda/Swahili (17.4, 95% CI 17.0–17.8). In the analysis limited to 15–24 year old men, there was no difference by ethnic group in the age at first sex in men. Among 15–49 year old women, there was a high degree of variation in mean age at first sex between ethnic groups. In all surveys, Luo women reported a younger age at first sex. In the early surveys (1989–1998), the reported average for Luo women was around 15 years old, while in many other groups the reported average was between 16 and 17 years old. Overall HIV prevalence was associated with lower age at first sex, by ethnic group. However, only two associations were significant at lower than P = 0.1∶1998 survey r = −0.68, P = 0.04; 2008 survey r = −0.62, P = 0.05. Among 15–24 year old women, negative associations were found in all surveys, with statistical significance below P = 0.1 also found in the 1993 survey: r = −0.68, P = 0.04; and 1998 survey: r = −0.73, P = 0.02.

#### Premarital sex

For both women and men, the Luo were more likely to report premarital sex than a number of the other groups. Among men, the contrast was greatest in the 2008 survey when 70.0% (95% CI, 63.6.5–76.4%) of the Luo versus 12.8% (95% CI, 3.5–22.1%) of the Somali reported premarital sex. In the women, this contrast was greatest between the Somali (1.9%, 95% CI 0.0–3.9%), Mijikenda/Swahili (27.1%, 95% CI 20.5–33.8%) and the Luo (57.8%, 95% CI 51.0–64.7%). The correlation analysis showed a fairly weak positive association between HIV prevalence and premarital sex among men. The strength of the relationship was much stronger among women with a fairly strong positive association ranging between 0.59 and 0.64 in the different surveys (p-values either 0.08 or 0.09).

#### Number of sex partners

There was significant variation in mean number of lifetime sexual partners between ethnic groups among men and women. In 2008, the Somali reported the lowest mean number of lifetime sex partners (men: 2.4, 95% CI 1.6–3.2; women: 1.1, 95% CI 1.0–1.2), the Kikuyu the highest amongst the men (8.5, 95% CI 7.1–10.0) and the Kamba the highest amongst the women (2.6, 95% CI, 2.4–2.8). In a number of groups there was a significant reduction in the percent of men and women reporting two or more partners and in the mean number of sexual partners compared to data from earlier surveys. There was a fairly strong positive association between the number of partners and HIV prevalence for men or women, but these relationships were not statistically significant. This was true for the proportion with two or more partners in the past year, the mean number of partners over the past year and the mean number of lifetime partners.

#### Sex with a non-married, non-cohabiting partner

In both the women and the men, the Somali had the lowest prevalence of sex with a non-married, non-cohabiting partner (men: 7.7%, 95% CI 0.9–14.6%; women: 1.2%, 95% CI 0.3–2.7%) and the Luos the highest prevalence (men: 40.2%, 95% CI 33.2–47.1%; women: 23.8%, 95% CI 20.0–28.1). This factor was only assessed in the 2008 survey. There was a non-significant trend for those ethnic groups with higher HIV prevalence to be more likely to report sex with a non-married, non-cohabiting partner (women: r = 0.60, P = 0.09; men r = 0.54, P = 0.12).

#### Polygamy

The prevalence of polygamy was low in the Kamba (2.8%, 95% CI 1.6–4.1), Kikuyu (3.8%, 95% CI 1.8–5.9) and Meru (5.4%, 95% CI 2.5–8.3) and high in the Luhya (20.9%, 95% CI 14.4–27.5), Luo (22.1%, 95% CI 17.5–26.8) and Somalis (30.5%, 95% CI 23.5%–37.6%; all from the 2008 survey). There was a weak and non-significant positive correlation between the prevalence of polygamy and HIV prevalence.

#### Cumulative prevalence of concurrency

The prevalence of concurrency was higher in men than women and varied significantly by *province*. The prevalence of male concurrency in Nyanza province (21.1%, 95% CI 17.2–24.9%), where the Luo predominate, was significantly higher than in the East Province (7.8%, 95% CI 5.0–10.7%). We found a positive association between the prevalence of HIV and male concurrency (Spearman’s rho = 0.79, P = 0.04).

#### Age gap

There was no association between the proportion of women who had a partner more than 5 years older than them and HIV prevalence by ethnic group (r = 0.00, P = 0.99).

#### Condom usage

Condom usage was fairly low in all ethnic groups in the 1989 GPA data. The GPA survey found that the proportion of men in each ethnic group who reported ever having used a condom ranged from 12.5 to 28.6%; median 17.9%. In the 2003 survey, respondents were asked if they had used a condom in the past 6 months. The proportion of men in each group that answered yes ranged from 13.1 to 28.5%; median 17.2%. By 2008, condom usage (measured as condom usage at last sex by men) had become more common among the most HIV affected ethnic groups. The Luo reported the highest rate of usage at last sex (40.8%: 95% CI 34.2–47.3). There was a trend for ethnic groups who in 2008 had higher HIV prevalence to report higher condom usage in 1989 and 2008.

#### Cultural acceptance of male concurrency

Amongst women the belief that it was culturally acceptable for a man to have more than one partner ranged between 22.2% (CI 15.1–29.3) in the East to 60.5% (CI 53.1–67.8%) in Nyanza province and 61.5% (CI 50.6–72.4) in the Western province. There was a positive association between this variable in women and HIV prevalence by province (r = 0.69; P = 0.08). The strength of this relationship was much weaker in men (r = 0.33; P = 0.46). The results were substantively similar when we restricted the analyses to the 15–25 year old respondents.

#### Approval of polygamy

In four groups (Kalenjin, Kamba, Kisii, Kikuyu) few men approved of polygamy (10–13%) whereas in the Luo, Luhya and Mijikenda polygamy was looked upon more favourably (31.0, 42.7 and 63.6% approving of polygamy, respectively). A very weak positive association was found between approval of polygamy in 1989 and HIV prevalence in 2008.

#### STI treatment

The percent of those with STI symptoms who went for STI treatment to a medical centre did not vary much by ethnic group and was not associated with HIV prevalence (r = 0.12; P = 0.77).

#### Ever tested

Ethnic groups with higher HIV prevalence tended to have a higher percentage of members who reported that they had been for an HIV test (men: r = 0.68, P = 0.04; women r = 0.61, P = 0.07). In both men and women, the Luo reported the highest and the Somali the lowest testing rates.

#### Sensitivity analyses

Sensitivity analyses using (1) the HIV prevalence rates by ethnic group from the 2003 KDHS as the dependent variable; and (2) the average of the 2003 and 2008 KDHS’s HIV prevalence rates by ethnic group as the dependent variable did not affect the results (data not shown).

## Discussion

If Kenya’s ethnic groups were countries then the Luo and the Somali would have the 5^th^ and the 60^th^ highest HIV prevalence in the world (based on 2008 prevalence) [1], [19]. Establishing what is responsible for these differences in HIV prevalence would provide useful information for programming and policies to reduce HIV infection in the most affected groups. According to the published literature a number of risk factors may have been responsible for these variations in HIV prevalence. The high rates of mobility of the fisherman and women trading fish in the Lake Victoria area as well as the exchange of “sex-for-fish” have been argued to play a role in the severity of the Nyanza epidemic [20]–[22]. In addition, the lower circumcision rates and the practice of wife inheritance amongst the Luo may have played a role in this group [23].

Studies from a range of locations have demonstrated that sexual partnering often tends towards a high degree of homophilous partnering by ethnicity [2]–[5]. Consistent with these studies, we found strong evidence of ethnic homophilous partnering in Kenya from data collected early in the epidemic and more recently. This indicates that differences in sexual mixing patterns between different ethnic groups may explain HIV variation between groups. Evidence that population differences in susceptibility alleles are able to explain the large differences in HIV prevalence between populations has not been found [11], [24]. Of note, while the Kenyan ethnic group with the highest HIV prevalence (the Luo) are a Nilotic language, so too are the Kalenjin who in 2008 had the second lowest HIV prevalence. Genetic susceptibility is therefore a less likely explanation for differences in HIV prevalence.

Differential circumcision almost certainly plays an important role in explaining the higher HIV prevalence in the Luo. The prevalence of circumcision amongst the Luo is, however, higher than at least 58% of countries with available data – most of whom have HIV prevalences less than 1% [7], [25], [26]. Furthermore, despite near universal male circumcision coverage in the non-Luo groups, there was considerable variation in HIV prevalence between these groups. The Luhya, for example, have a slightly higher circumcision prevalence than the Kalenjin, but an HIV prevalence more than double that of the Kalenjin. Likewise, the prevalence of HIV is nine times higher in the Luhya than the Somalis despite similar circumcision rates. Therefore, while circumcision plays a major role it is likely that other factors contribute to between group differences.

Previous analysis of factors associated with HIV infection in the 2003 KDHS concluded that “sex-related behavioral factors did not have as great an impact in the analyses as expected and… that differences in biological factors such as circumcision and sexually transmitted infections may be more important in assessing risk for HIV than differences in sexual behavior [27].” Controlling for circumcision the study found that for both men and women, the second most strongly associated risk factor was being resident in Nyanza Province. What could explain this finding? Like others, Johnson et al. speculate that differences in STI prevalence may be important. Understanding what explains the higher STI rates could therefore help elucidate mechanisms behind differential HIV prevalence. Were differences in STI rates due to variation in sexual behavior or perhaps differences in the efficacy of STI treatment? In our study we did not find evidence that differences in STI treatment-seeking-behavior was associated with HIV prevalence.

Differences in sexual behavior constitute an alternative set of factors that could contribute to the explanation for the variations in HIV prevalence. Our study finds evidence of considerable variations in sexual behavior between ethnic groups in Kenya. These include the age at first sex, number of sexual partners, the prevalence of concurrent sexual partnerships, premarital sex, sex with a non-marital or non-cohabiting partner and condom use. Each of these factors was found to be correlated with HIV prevalence, although the relationship was not always found to be statistically significant.

The positive association between condom use in 2008 and HIV prevalence likely reflects the case that condoms are more likely to be used in higher prevalence areas. Given the positive correlation between HIV testing and HIV prevalence a significant proportion of condom use in higher prevalence areas will likely consist of protected sex among individuals who know they are living with HIV. Our finding that the Luo, who in 2008 had the highest HIV prevalence, had the highest rate of condom use early in the epidemic (1989) could indicate that sexual behaviors perceived as risky for STI (as proxied by condom use) were more common in the Luo early in the HIV epidemic. It could also mean that family planning was a greater priority in areas inhabited among the Luo and that low rates of male circumcision and other sexual risk behaviors lead to higher HIV prevalence in this group despite consistent higher levels of condom use.

The contribution that age at first sex and number of sexual partners play in exposure to HIV is well established. Similar associations between HIV risk and premarital sex and sex with a non-cohabiting partner have been found in previous studies [9], [28]. It is not however clear what these factors are a proxy for and further research is required to examine the mechanisms underlying these associations. The positive correlation between male concurrency and HIV prevalence found in our study may indicate that differences in sexual network structure contribute to ethnic differentials in HIV. Furthermore, a strong positive association was found between HIV prevalence and women’s beliefs that concurrency is culturally acceptable and we found that a high proportion of the newly sexually active (15–25 year olds) regard multiple partnering as culturally acceptable.

Sexual concurrency has been shown to link up sexual networks in a way that can lead to the rapid and extensive spread of STIs in affected groups [2]. A number of studies have found evidence that differences in sexual network structure in general, and network connectivity in specific, play an important role in determining the large ethnic differences in STI prevalence seen in other countries such as the USA [7], [25], [26], Canada [7], [8], the UK [7]–[9], [29], [30] and South Africa [6], [7], [31]. There are many complex reasons for why it is difficult to find evidence for or against the link between concurrency and HIV infection risk or prevalence [32] Many individual-level risk factor analyses have been attempted, but as HIV risk due to concurrency operates at the level of networks, network-level factors need to be assessed at a population level. Although we found a positive association between concurrency and HIV prevalence at a population level, this association could be explained by the fact that concurrency and numbers of sexual partners are highly correlated and we could not isolate the effect of these factors.

We found no evidence of a statistically significant relationship between polygamy and HIV prevalence. Of note the highest prevalence of polygamy was in the Somalis, the group with the lowest HIV prevalence. Polygamy is a form of concurrency that can be considered a feature of a closed sexual network (if all married parties refraining from extramarital sex) and therefore a type of concurrency that may not be expected to enhance HIV spread [33], [34].

There are a number of limitations with this study. The surveys between 1989 and 1998 were not HIV serolinked and thus the behaviors (aggregated into ethnic groups) in these surveys are compared with HIV prevalence from the 2008 survey. Utilizing a series of cross-sectional surveys that use slightly different sampling and questioning strategies is suboptimal. The surveys were not designed to generate samples that were representative of the individual ethnic groups. Some of the ethnic group sample sizes are small and these provide unstable prevalence estimates for both the dependent and independent variables. Furthermore, the DHS and GPA surveys used suboptimal methodologies to determine sensitive information such as concurrent partnering [35]. Some of the surveys had low response rates (see Table 1). The data is thus susceptible to a large number of biases such as courtesy, recall and nonresponse biases. There is however little evidence that we are aware of that there is a difference in sexual behavior between those who do and do not answer sexual behavior questionnaires [40]. Unfortunately the response rates by ethnic group are not reported in any of the surveys we used but the HIV Testing response rate did vary by ethnic group. The analysis is explicitly ecological in nature, which makes any inference to the individual level inappropriate. In addition, the relationships between sexual behavior, ethnic group and HIV prevalence could be confounded by other unmeasured variables. One such factor could be ethno-linguistic heterogeneity which has been shown in a four country study (that included Kenya) to be positively related to risky sexual behavior and the HIV status of individuals [36]. Given the low prevalence of antiretroviral coverage over the time course of the study (less than 10% in 2005 and 42% in 2007 of those eligible for antiretroviral therapy were receiving it) we regard it as unlikely that antiretroviral therapy influenced our results [37], [38].

The form of concurrent partnering that has been most closely linked with the generation of generalized HIV epidemics is the practice of having side-partners (often secret) alongside main-partners [39]. Unfortunately the methodologies used in the GPA and DHS surveys have been shown to be suboptimal in terms of eliciting sensitive sexual behavior data, such as this form of concurrent partnering [35].

Overall, this study suggests that the large variations in HIV prevalence in Kenya’s ethnic groups may be partly explained by both differences in male circumcision prevalence and variation in sexual behavior. While the roll-out of medical male circumcision and antiretroviral treatment are major components of the HIV prevention strategy in high prevalence areas, such as Nyanza province, our study indicates that interventions to reduce risky sexual behavior should continue to be considered as complements to biomedical and structural intervention efforts. In particular, our study suggests that concurrency at the population level may play a role. Future research should examine the link between concurrency and HIV at the population level and assess the relationship between beliefs about the cultural acceptability of concurrency and actual behavior and HIV risk.
